# Epidemiology of *Taenia saginata* taeniosis/cysticercosis: a systematic review of the distribution in southern and eastern Africa

**DOI:** 10.1186/s13071-018-3163-3

**Published:** 2018-11-06

**Authors:** Veronique Dermauw, Pierre Dorny, Uffe Christian Braae, Brecht Devleesschauwer, Lucy J. Robertson, Anastasios Saratsis, Lian F. Thomas

**Affiliations:** 1 Department of Biomedical Sciences, Institute of Tropical Medicine, Antwerp, Belgium; 2 Department of Virology, Parasitology and Immunology, Faculty of Veterinary Medicine, Ghent University, Merelbeke, Belgium; 3 One Health Center for Zoonoses and Tropical Veterinary Medicine, Ross University School of Veterinary Medicine, Basseterre, Saint Kitts Trinidad and Tobago; 4 Department of Epidemiology and Public Health, Sciensano, Brussels, Belgium; 5 Department of Veterinary Public Health and Food Safety, Faculty of Veterinary Medicine, Ghent University, Merelbeke, Belgium; 6 Parasitology, Department of Food Safety and Infection Biology, Faculty of Veterinary Medicine, Norwegian University of Life Sciences, Adamstuen Campus, Oslo, Norway; 7 Laboratory of Parasitology, Veterinary Research Institute, Hellenic Agricultural Organization Demeter, Thermi, 57001 Thessaloniki, Greece; 8 International Livestock Research Institute (ILRI), P.O. Box 30709, Nairobi, Kenya; 9 Institute for Infection and Global Health, University of Liverpool, Neston, UK

**Keywords:** *Taenia saginata*, Cestode, Beef tapeworm, Taeniosis, Bovine cysticercosis, Eastern Africa, Southern Africa

## Abstract

**Background:**

The beef tapeworm, *Taenia saginata*, causing cysticercosis in bovines and taeniosis in humans, is thought to have a global distribution. In eastern and southern Africa, cattle production plays a crucial role in the economy, but a clear overview of the prevalence of *T. saginata* in the region is still lacking. This review aims to summarize existing knowledge on *T. saginata* taeniosis and bovine cysticercosis distribution in eastern and southern Africa.

**Methods:**

A systematic review was conducted, that gathered published and grey literature, including OIE reports, concerning *T. saginata* taeniosis and bovine cysticercosis in eastern and southern Africa published between January 1st, 1990 and December 31st, 2017.

**Results:**

A total of 1232 records were initially retrieved, with 78 full text articles retained for inclusion in the database. Unspecified taeniosis cases were reported for Angola, Ethiopia, Kenya, Madagascar, Malawi, South Africa, Tanzania, Uganda and Zambia, whereas *T. saginata* taeniosis cases were found for Ethiopia, Kenya, South Africa, Tanzania, Zambia and Zimbabwe. The prevalence of taeniosis ranged between 0.2–8.1% based on microscopy, and between 0.12–19.7% based on coproAg-ELISA. In Ethiopia, the percentage of tapeworm self-reporting was high (45.0–64.2%), and a substantial number of anthelmintic treatments were reported to be sold in towns. The presence of bovine cysticercosis was reported in all 27 countries/territories included in the study, except for Rwanda and Somalia, Comoros, Madagascar, Mauritius, Mayotte, Seychelles and Socotra. The prevalence of cysticercosis ranged between 0.02–26.3% based on meat inspection, and between 6.1–34.9% based on Ag-ELISA.

**Conclusions:**

Although *T. saginata* has been reported in the majority of countries/territories of the study area, *T. saginata* taeniosis/cysticercosis remains a largely ignored condition, probably due to the absence of symptoms in cattle, the lack of data on its economic impact, and the fact that human taeniosis is considered a minor health problem. However, the occurrence of bovine cysticercosis is a clear sign of inadequate sanitation, insufficient meat inspection, and culinary habits that may favour transmission. Measures to reduce transmission of *T. saginata* are therefore warranted and the infection should be properly monitored.

**Electronic supplementary material:**

The online version of this article (10.1186/s13071-018-3163-3) contains supplementary material, which is available to authorized users.

## Background

The beef tapeworm, *Taenia saginata*, utilizes bovines as intermediate hosts and humans as final hosts. Although tapeworm infections have been reported since ancient times [1], it was not until 1782 [2] that differentiation of *T. saginata* from the other well-known meat-transmitted human tapeworm, *Taenia solium*, was established. Furthermore, it was not until 1871 that the role of cattle as intermediate hosts for the parasite was established, with “measly” beef being reported as the source of infection in patients [3].

Ingestion of raw or undercooked infected beef is indeed the mode of transmission of this zoonotic parasite to humans, in whom it develops to its adult form, a several metres long segmented worm consisting of a scolex with four suckers, neck and strobila, i.e. a chain of proglottids [4]. In contrast to *T. solium*, the gravid proglottids of *T. saginata*, which contain thousands of embryonated eggs, are mobile and can migrate from the anus independently of, as well as during, defaecation [5]. Eggs are then shed into the environment, and cattle become infected through grazing contaminated pastures, or ingesting contaminated fodder or water. After hatching, and penetration of the intestinal wall, the oncospheres reach the general circulation, distributing them throughout the body where they develop into cysticerci [4]. Common predilection sites for *T. saginata* cysticerci include the heart and masseter muscles [6].

In both intermediate and definitive hosts, *T. saginata* causes few symptoms. In humans, infection is usually characterized by anal pruritus due the active migration of *T. saginata* proglottids and some mild abdominal pain [7]. Nevertheless, the (potential) presence of a tapeworm in the body can cause distress [8], and some people even suffer from a pathological fear of tapeworms, often encouraged by horror stories circulating in popular media or books [9, 10]. Moreover, although rare, complications due to taeniosis, such as appendicitis, have been reported [11]. In cattle, the infection is generally asymptomatic but nevertheless may incur great economic losses for the meat sector due to carcass condemnation or treatment upon detection of cysticerci during meat inspection, as well as related insurance costs [12, 13].

*Taenia saginata* is distributed globally, with the parasite occurring in both developed and developing countries, although less frequently in countries where cultural preferences limit consumption of bovids or where adequate sanitary infrastructure reduces the likelihood of bovids ingesting human faecal matter. Thus, the prevalence of human taeniosis and bovine cysticercosis are considered particularly high in Africa, Latin America and some parts of Asia [4].

In eastern and southern Africa, the cattle population was estimated at a massive 20.6 million in 2016 [14], so the parasite is thought to be of particular relevance here. In the area, bovines are essential for the livelihoods of smallholders, serving as a source of food, draft power and manure, as well as acting as a financial buffer for challenging times. Although there are indications of the widespread presence of the parasite in at least some countries in this region (e.g. Ethiopia: [15–17]), an extensive overview of its distribution in this region, along with epidemiological considerations regarding its presence, is still lacking. Our aim was therefore to gather recent information on the presence of *T. saginata* in eastern and southern Africa.

## Methods

### Search strategy

A systematic review of published literature was conducted to collect data on the occurrence, prevalence, and geographical distribution of bovine cysticercosis and human taeniosis in eastern and southern Africa, published between January 1st, 1990 and December 31st, 2017. For the purpose of this study, eastern and southern Africa was defined as the area covered by the following countries/territories: Angola, Botswana, Burundi, Comoros, Djibouti, Eritrea, Ethiopia, Kenya, Lesotho, Madagascar, Malawi, Mauritius, Mayotte (French), Mozambique, Namibia, Réunion (French), Rwanda, Seychelles, Socotra (Yemini), Somalia (including the autonomous regions Puntland and Somaliland), South Africa, Swaziland, Tanzania (including the semi-autonomous region of Zanzibar), Uganda, Zambia and Zimbabwe. The PRISMA guidelines were followed whilst conducting the review [18] (Additional file 1). The search protocol can be found in Additional file 2.

The international bibliographic databases PubMed (http://www.ncbi.nlm.nih.gov/pubmed) and Web of Science (http://ipscience.thomsonreuters.com/product/web-of-science/) were searched using the following search phrase: (cysticerc* OR cisticerc* OR “C. bovis” OR taenia* OR tenia* OR saginata OR taeniosis OR teniosis OR taeniasis OR ténia OR taeniid OR cysticerque) AND (Angola OR Botswana OR Burundi OR Comoros OR Djibouti OR Eritrea OR Ethiopia OR Kenya OR Lesotho OR Madagascar OR Malawi OR Mauritius OR Mayotte OR Mozambique OR Namibia OR Réunion OR Rwanda OR Seychelles OR Socotra OR Somalia OR South Africa OR Swaziland OR Tanzania OR Uganda OR Zanzibar OR Zambia OR Zimbabwe OR “East Africa” OR “Horn of Africa” OR “Southern Africa” OR Puntland OR Somaliland).

Furthermore, a range of databases for grey literature and MSc/PhD thesis documents were searched using keywords from the above search phrase (the full list of databases is presented in Additional file 3). Data on bovine cysticercosis from the different scientific databases were complemented with data from OIE databases “Handistatius” (1996–2004) and “WAHIS” (2005) [19, 20]. Finally, reference lists of reviews on the topic were screened and additional relevant records were added to the database.

### Selection criteria

Upon compilation of search results from the different databases, duplicate records were removed. Thereafter, titles and abstracts were screened for relevance, applying the following exclusion criteria: (i) studies concerning a parasite other than *T. saginata*; (ii) studies conducted outside the study area; (iii) studies published outside the study period; (iv) studies reporting results outside the scope of the review question (e.g. review, experiment, intervention trial); and (v) duplicated data. After the screening process, full text articles were evaluated using the same criteria listed above (Additional file 4).

### Data extraction and generation

Data from included records were extracted. In reports where the numerator and denominator of the study sample were available, prevalence data were calculated, if not already provided. When not presented in the manuscript, the 95% exact confidence intervals (CI) were calculated, using the “binom.test” function (“*stats*” package) in R 3.5.1 [21].

## Results

### Search results

A total of 1228 records were obtained from the database search, and four additional records were added through screening of the reference lists of relevant reviews (Additional file 4). After removal of duplicate records (*n* = 71), 1161 records were screened based on title and, thereafter, abstract. During title screening, 987 records were excluded, and a further 85 records were removed upon abstract screening; three of these were remaining duplicate records, whereas the other removed records focussed on a different parasite (*n* = 32) or study area (*n* = 18), were published outside the study period (*n* = 1), or had a different scope (e.g. laboratory experiments, review) (*n* = 31). Thus, 89 full text articles (*n* = 89) fulfilled the eligibility criteria for evaluation, but three of these were unavailable. During the evaluation of the remaining 86 records, eight were excluded due to having a different scope.

Thus, 78 records were included in the qualitative synthesis (journal articles: 73, online data repositories: 2, MSc thesis: 2, PhD thesis: 1). Apart from the two OIE sources describing the occurrence of bovine cysticercosis throughout the study area, the majority of records presented data from Ethiopia (*n* = 37). The others included data from Kenya (*n* = 11), Tanzania (*n* = 7), South Africa (*n* = 7), Zambia (*n* = 4), Zimbabwe (*n* = 2), Angola (*n* = 2), Uganda (*n* = 1), Swaziland (*n* = 1), Namibia (*n* = 1), Malawi (*n* = 1), Madagascar (*n* = 1) or Botswana (*n* = 1).

### Human taeniosis occurrence

**Fig. 1:**
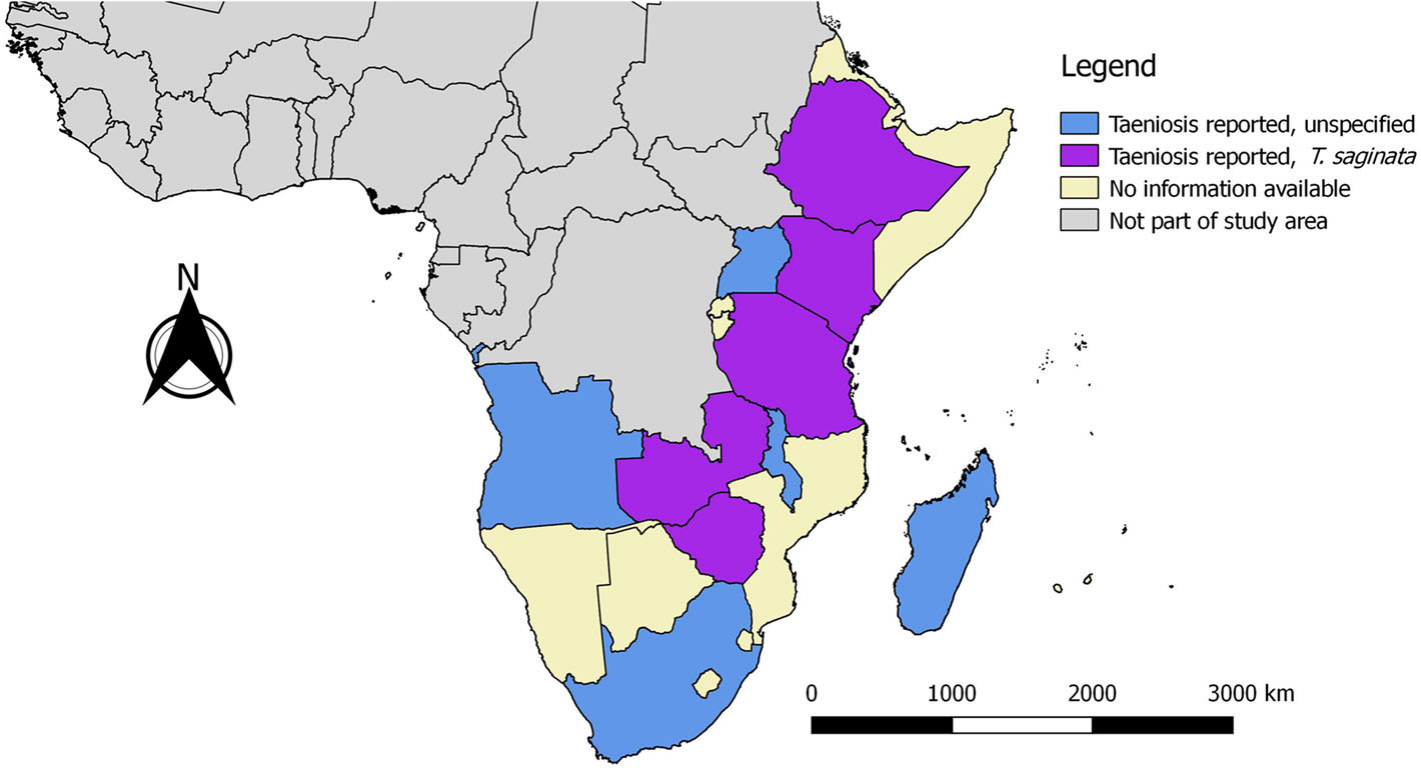
Human taeniosis in southern and eastern Africa

A total of 48 records reported the presence of human taeniosis cases (excluding those with confirmed *T. solium* taeniosis). Unspecified taeniosis cases were reported from Angola, Ethiopia, Kenya, Madagascar, Malawi, South Africa, Tanzania, Uganda and Zambia, whereas known *T. saginata* taeniosis cases were reported from Ethiopia, Kenya, South Africa, Tanzania, Zambia and Zimbabwe (Fig. 1). Microscopy results were included in 32 reports, most of which presented data from Ethiopia (18) (Table 1). Taeniosis prevalence based on microscopy alone ranged between 0.2–8.1% (villagers in Kenya [22] and Ethiopia [23], respectively), and one study reported the absence of taeniosis (in geophagous pregnant women in Kenya [24]). Four records presented data from coproAg-ELISA studies conducted in Kenya and/or Zambia, with a prevalence ranging between 0.12–19.7% (villagers in Zambia [25] and Kenya [26], respectively) (Table 2), two of which involved confirmed *T. saginata* cases. Overall, common study groups were school-children, patients suffering from other diseases [e.g. HIV infection, sleeping sickness and active pulmonary tuberculosis (TB)], as well as occupational groups (e.g. tobacco farm workers, food handlers). Furthermore, eight studies reported taeniosis prevalence in communities based on self-reporting by questionnaire respondents (prevalence range: 45.5–64.2%) (Table 3), and five records presented data on anthelmintic sales in towns (Table 4), both in Ethiopia. Another two records contained data on household latrine sampling, thus presenting prevalence at the household level (Malawi: 40.4% [27]; South Africa: 18.0% [28]). Finally, one report discussed a case of intestinal obstruction due to impaction of a *T. saginata* tapeworm in Zimbabwe, requiring enterotomy with bolus removal as well as appendectomy [29].

**Table 1:** Reported occurrence of taeniosis in southern and eastern Africa: microscopy studies

Country	Period	People tested	People positive	Prevalence (%) (95% CI)	Species identification	Group studied	Reference
Angola	9/2012-12/2013	344	2	0.58 (0.07–2.1)	N	Children below 5 with diarrhea	[50]
Angola	1/2015-5/2015	230	2	0.87 (0.10–3.1)	N	School children in 16 schools	[51]
Ethiopia	11/1995-4/1996; 6-9/1996	1750	79	4.5 (3.6–5.6)	Y^a^	Sugar-estate residents	[52]
Ethiopia	3-4/1999; 2/2002	3167	na	< 4 (na)	N	Schoolchildren, peasants and teachers	[53]
Ethiopia	1/2002-2/2002	104	1	0.96 (0.02–5.2)	Y^a^	HIV/AIDS and HIV-seronegative individuals in a teaching hospital	[54]
Ethiopia	2007–2012	32191	322	1.0 (0.9–1.1)	N	Rural hospital visiters	[55]
Ethiopia	5/2007-6/2007	419	na	1.4 (na)	N	na	[56]
Ethiopia	12/2007-2/2008	7171	23	0.32 (0.20–0.48)	N	Visitors of health centers	[57]
Ethiopia	8/2008-12/2008	343	14	4.1 (2.2–6.8)	N	HIV patients recruited at hospital	[58]
Ethiopia	11/2008	121	5	4.1 (1.4–9.4)	N	Prison inmates	[59]
Ethiopia	11/2008	115	1	0.87 (0.02–4.7)	N	Tobacco farm workers	[59]
Ethiopia	4/2009	384	5	1.3 (0.4–3.0)	N	Food handlers	[60]
Ethiopia	9/2010-7/2011	858	18	2.1 (1.2–3.3)	N	Highland and lowland dwellers	[61]
Ethiopia	1/2011-6/2011	200	1	0.5 (0.01–2.8)	N	Food handlers	[62]
Ethiopia	3/2012-11/2012	260	1	0.38 (0.01–2.12)	N	Children recruited in Health Center	[63]
Ethiopia	1/2013-5/2013	172	5	2.9 (1.0–6.7)	N	Asymptomatic food handlers	[64]
Ethiopia	8/2013-11/2013	180	2	1.1 (0.13–4.0)	N	HAART initiated and naive paediatric HIV patients	[65]
Ethiopia	1/2015-2/2015	503	13	2.6 (1.4–4.4)	N	School children from 5 schools	[66]
Ethiopia	1/2016-8/2016	213	5	2.3 (0.8–5.4)	N	Active pulmonary TB patients	[67]
Ethiopia	na	1537	na	8.1 (na)	Y^a^	Participants from 19 communities, includes children and adults	[23]
Ethiopia	na	491	12	2.4 (1.3–4.2)	N	Villagers	[68]
Kenya	2000–2009	31	1	3.2 (0.08–16.7)	N	Sleeping sickness patients	[69]
Kenya	7/2010-7/2012	2057	na	0.20 (na)	N	na	[22]
Kenya	8/2010-7/2012	2113	na	0.30 (0–0.5)	N	Mixed-farming community	[26]
Kenya	na	285	na	5.3 (na)	N	HIV-positive patients	[70]
Kenya	na	151	0	0 (0–2.4)	N	Geophagous pregnant women	[24]
Madagascar	11/1996-1/1997	401	3	0.75 (0.15–2.2)	N	Patients referred for parasitological examination	[71]
South Africa	2009	na	2	na	N	Laboratory results	[72]
South Africa	2009	na	4	na	Y	Laboratory results	[72]
South Africa	4/2009-9/2009	162	3	1.9 (0.4–5.3)	N	School children	[73]
South Africa	2010	na	11	na	Y	Laboratory results	[72]
South Africa	2010	na	1	na	N	Laboratory results	[72]
South Africa	na	183	3	1.6 (0.3–4.7)	N	Rural black preschool children	[74]
Tanzania	2008–2009	1057	3	0.30 (0.06–0.8)	Y^b^	Villagers, after treatment with niclosamide/praziquantel and purgation	[75]
Uganda	na	5313	36	0.70 (0.5–0.9)	N	Primary school children	[76]
Zambia	6/2007-8/2007	403	na	0.90 (na)	N	School children	[77]

^a^Reported as *T. saginata*, yet unclear from methodology

^b^Confirmed by PCR

*Abbreviations*: CI, confidence interval; na, not available; HAART, highly active antiretroviral therapy

**Table 2:** Reported occurrence of taeniosis in southern and eastern Africa: coproAg-ELISA studies

Country	Study period	People tested	People positive	Prevalence (%) 95% CI	Species identification	Group studied	Reference
Kenya	1/2007-4/2007	204	12	5.9 (3.1–10.0)	Y^a^	District inhabitants	[78]
Kenya	8/2010-7/2012	2113	na	19.7 (16.7–22.7)	N	Mixed-farming community	[26]
Kenya	na	691	na	1.9 (na)	N	Slaughterhouse workers	[79]
Zambia	2006	190	5	2.6 (0.9–6.0)	Y^a^	Pupils primary schools	[78]
Zambia	8/2009;10/2010	817	1	0.12 (0.003–0.68)	Y^b^	Consenting villagers	[25]

^a^Results based on coproAg-ELISA, specific for *T. saginata*

^b^Results based on coproAg-ELISA with coproPCR (*T. saginata* specific) confirmation

*Abbreviations*: CI, confidence interval; na, not available

**Table 3:** Reported occurrence of taeniosis: questionnaire studies in Ethiopia

Town	Study period	People interviewed	People reporting infection	Prevalence (%) 95% CI	Reference
Awassa	10/2005-4/2006	120	77	64.2 (54.9–72.7)	[44]
Soddo	11/2007-4/2008	79	40	50.6 (39.1–62.1)	[45]
Jimma	11/2008-3/2009	60	34	56.7 (43.2–69.4)	[46]
Yirgalem	11/2009-3/2011	170	119	70.0 (62.5–76.8)	[47]
Sebeta, Tulu Bolo, Weliso	na	392	na	55.1 (na)	[48]
Harar	na	300	182	60.7 (54.9–66.2)	[16]
Adama	11/2013-4/2014	200	91	45.5 (38.5–52.7)	[15]
Batu	12/2014-4/2015	100	59	59.0 (48.7–68.7)	[49]

*Abbreviations*: CI, confidence interval; na, not available

**Table 4:** Reported town level taeniicidal sales in Ethiopia

Town	Year	Number	Value (ETB)	Value (EUR)^a^	Reference
Awassa	2002	1,582,254	1,880,330	58,290	[44]
Awassa	2003	1,221,004	1,746,585	54,144	[44]
Awassa	2004	946,330	1,803,300	55,902	[44]
Awassa	2005	889,759	1,788,776	55,452	[44]
Soddo	2004	74,747	192,979	5982	[45]
Soddo	2005	77,705	203,675	6342	[45]
Soddo	2006	79,230	210,133	6314	[45]
Soddo	2007	105,090	279,660	8669	[45]
Jimma	2007	51,462	na	na	[46]
Jimma	2008	52,134	na	na	[46]
Yirgalem	2005	95,712	na	na	[47]
Yirgalem	2006	93,059	na	na	[47]
Yirgalem	2007	95,093	na	na	[47]
Yirgalem	2008	95,121	na	na	[47]
Yirgalem	2009	93,028	na	na	[47]
Batu	2013	42,557	148,100	4591	[49]
Batu	2014	29,049	97,492	3022	[49]

^a^Based on July 2018 exchange rates (1 ETB = 0.0310 EUR)

*Abbreviation*: na, not available

### Bovine cysticercosis

**Fig. 2:**
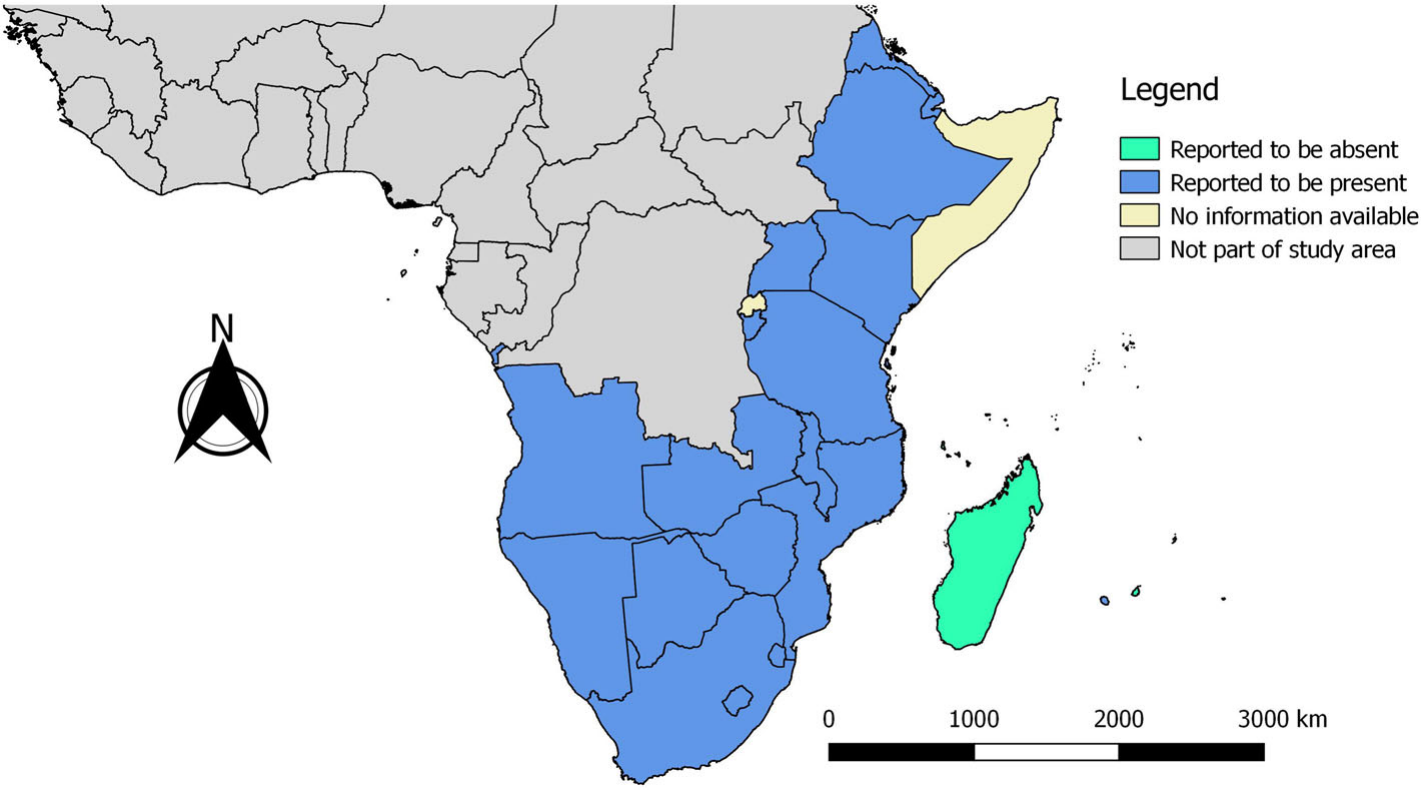
Bovine cysticercosis in southern and eastern Africa

Based on the retrieved data sources (both OIE databases and manuscripts/reports), the presence of bovine cysticercosis was reported in all of the 27 countries/territories studied, except for Comoros, Madagascar, Mauritius, Mayotte and Seychelles. In addition, no information was available for Rwanda, Somalia, Mayotte and Socotra (Fig. 2). Data from the two OIE data sources indicating the occurrence and/or number of cases are presented in Table 5. Apart from the OIE data sources, a total of 39 records were found to document results on bovine cysticercosis in the study region. Meat inspection results were included in 35 records (Table 6), with prevalence estimates ranging between 0.02–26.3%, while two records reported the absence of positive animals (Tanzania: 2011 [30], Zambia: 2001 [31]). Seven records provided serological data, mostly based on Ag-ELISA results (prevalence range: 6.1–53.5%), while one presented Ab-ELISA data (prevalence: 10.0%) [32] and another IHAT results (prevalence: 25.7%) [33] (Table 7). One study estimated the town level costs due to condemnation caused by bovine cysticercosis [Mekelle, abattoir level: 31,952 ETB/6 months (991 EUR, according to July 2018 exchange rates; 1 ETB = 0.0310 EUR) [34]], and another five studies provided data on total economic losses due to condemnation for a wide variety of conditions [17, 30, 35–37]. Overall, the majority of records presented data from Ethiopia (21/41), followed by Tanzania (8/41) and Kenya (7/41).

**Table 5:** OIE data on occurrence of bovine cysticercosis in the southern and eastern Africa (1996–2005) [19, 20]

Country	Year									
	1996	1997	1998	1999	2000	2001	2002	2003	2004	2005
Angola	6	11	na	na	na	na	na	na	4	+
Botswana	na	53	4356	12,863	14,000	14,000	10,181	15,363	na	+
Burundi	na	na	na	na	na	+	na	na	na	na
Comoros	na	na	-	na	na	na	na	na	na	na
Djibouti	na	na	na	na	na	na	+	+	+	+
Eritrea	+	+	+	2	+	+	na	+	370	-
Ethiopia	+	na	na	na	na	+	+	+	+	+
Kenya	+	+	+	+	+	+	+	+	3999	na
Lesotho	56	38	139	na	na	na	68	na	na	-
Madagascar	na	na	na	na	na	na	-	na	na	-
Malawi	+	+	+	+	+	+	+	+	6	+
Mauritius	na	na	na	na	na	-	-	-	-	na
Mozambique	+	+	+	na	na	na	na	na	na	-
Namibia	+	3833	3118	+	2852	2226	2399	54	1616	+
Réunion	+	na	na	-	-	-	-	-	na	-
Rwanda	na	na	na	na	na	na	na	na	na	na
Seychelles	-	-	-	na	-	na	-	na	-	na
Somalia	na	na	na	na	na	na	na	na	na	na
South Africa	+	+	+	+	+	+	+	+	+	+
Swaziland	969	+	+	+	1419	530	245	1909	561	+
Tanzania	+	+	+	+	na	na	+	6	na	+
Uganda	+	24	152	+	+	+	+	na	na	+
Zambia	na	na	na	na	+	na	2	248	na	na
Zimbabwe	244	1744	2062	1988	160	1447	+	na	na	na

*Abbreviations*: na, not available; *+*, occurrence of the disease; *-*, absence of the disease

**Table 6:** Reported occurrence of bovine cysticercosis in southern and eastern Africa: meat inspection studies

Country	Period	Animals tested	Animals positive	Prevalence (%) (95% CI)	Reference
Botswana	1995	na	na	na (9.1–12.6)	[80]
Ethiopia	9/2004-8/2005	11,227	842	7.5 (7.0–8.0)	[81]
Ethiopia	9/2005-2/2007	4456	824	18.5 (17.4–19.7)	[82]
Ethiopia	10/2005-4/2006	400	105	26.3 (22.0–30.9)	[83]
Ethiopia	12/2006-7/2007	3711	308	8.3 (7.4–9.2)	[34]
Ethiopia	10/2007-3/2008	512	15	2.9 (1.6–4.8)	[84]
Ethiopia	11/2007-4/2008	415	47	11.3 (8.4–14.8)	[45]
Ethiopia	12/2007-2/2008	1023	74	7.2 (5.7–9.0)	[57]
Ethiopia	11/2008-3/2009	500	22	4.4 (2.8–6.6)	[46]
Ethiopia	10/2009-9/2010	898	177	19.7 (17.2–22.5)	[16]
Ethiopia	11/2009-3/2011	400	48	12 (9.0–15.6)	[47]
Ethiopia	2010	12,708	669	5.3 (4.9–5.7)	[37]
Ethiopia	9/2010-9/2012	3055	126 organs	na	[35]
Ethiopia	2011	34,674	3259	9.4 (9.1–9.7)	[37]
Ethiopia	2012	10,363	803	7.7 (7.2–8.3)	[37]
Ethiopia	10/2012-4/2013	745	21 organs	na	[35]
Ethiopia	2013	5172	247	4.8 (4.2–5.4)	[37]
Ethiopia	11/2013-4/2014	384	10	2.6 (1.3–4.7)	[15]
Ethiopia	11/2013-5/2014	3675	40 organs	na	[36]
Ethiopia	05/2014-6/2014	439	23	5.2 (3.3–7.8)	[85]
Ethiopia	12/2014-4/2015	384	10	2.6 (1.3–4.7)	[49]
Ethiopia	na	522	39	7.4 (5.4–10.1)	[33]
Ethiopia	na	1022	64	6.3 (4.9–7.9)	[17]
Ethiopia	na	1216	na	4.6 (na)	[48]
Kenya	1974	77,810	6784	8.8 (8.5–8.9)	[86]
Kenya	1975–1978	na	na	< 6.0 (na)	[86]
Kenya	1979–1983	na	na	< 4.0 (na)	[86]
Kenya	1984	na	na	1.8 (na)	[86]
Kenya	1985–1990	na	na	< 2.0 (na)	[86]
Kenya	1991	315,801	3457	1.1 (1.06–1.13)	[86]
Kenya	9/2006-1/2007	188	6	3.2 (1.2–6.8)	[87]
Kenya	na	511	39	7.6 (5.5–10.3)	[88]
Kenya	na	1184	189	16.0 (13.9–18.2)	[32]
Namibia	2000	12,204	973	8.0 (7.5–8.5)	[89]
Namibia	2001	7888	713	9.0 (8.4–9.7)	[89]
Namibia	2002	10,561	798	7.6 (7.1–8.1)	[89]
Namibia	2003	4411	347	7.9 (7.1–8.7)	[89]
Namibia	2004	5309	401	7.6 (6.9–8.3)	[89]
Namibia	2006	7085	435	6.1 (5.6–6.7)	[89]
Namibia	2006	71,388	243	0.34 (0.30–0.39)	[89]
South Africa	2009–2011	1,022,556	24,443	2.39 (2.36–2.42)	[90]
South Africa	2010	356,006	2169	0.61 (0.58–0.64)	[91]
South Africa	2011	349,458	2389	0.68 (0.66–071)	[91]
South Africa	2012	348,309	1980	0.57 (0.54–0.59)	[91]
South Africa	2013	361,232	3382	0.94 (0.91–0.97)	[91]
Tanzania	1987–1989	42,434	na	16.4 (na)	[92]
Tanzania	1/2002-4/2004	12,444	185	1.5 (1.3–1.7)	[93]
Tanzania	2005	na	19	0.06 (na)	[94]
Tanzania	2006	na	24	0.06 (na)	[94]
Tanzania	2007	na	16	0.04 (na)	[94]
Tanzania	2010	27,444	20 organs	na	[30]
Tanzania	2/2010-1/2011	30,713	18 organs	na	[95]
Tanzania	2011	30,671	0 organs	na	[30]
Tanzania	2012	27,865	6 organs	na	[30]
Tanzania	12/2013	2438	1 and 0.1% organs	na	[30]
Tanzania	na	na	21	na	[96]
Zambia	2000	4629	1	0.02 (0.0005–0.12)	[31]
Zambia	2001	9422	0	0 (0–0.04)	[31]
Zambia	2002	10,147	2	0.02 (0.002–0.07)	[31]
Zambia	2003	11,519	2	0.02 (0.002–0.06)	[31]
Zimbabwe	1/2006-12/2007	86,080	1364	1.6 (1.5–1.7)	[97]

*Abbreviations*: CI, confidence interval; na, not available

**Table 7:** Reported occurrence of bovine cysticercosis in southern and eastern Africa: serological studies

Country	Period	Diagnostic tool	Animals tested	Animals positive	Prevalence (%) 95% CI	Reference
Ethiopia	na	IHAT	743	190	25.6 (22.4–28.9)	[33]
Kenya	9/2006-1/2007	Ag-ELISA	188	44	23.4 (17.8–30.1)	[87]
Kenya	10/2006-11/2006	Ag-ELISA	792	143	18.1 (15.4–20.9)	[87]
Kenya	8/2010-7/2012	Ag-ELISA	983	na	53.5 (48.7–58.3)	[26]
Kenya	na	Ag-ELISA	511	117	22.9 (19.3–26.8)	[88]
Kenya	na	Ag-ELISA	1184	413	34.9 (32.2–37.7)	[32]
Kenya	na	Ab-ELISA	1184	118	10.0 (8.3–11.8)	[32]
South Africa	na	Ag-ELISA	1315	300	22.8 (20.6–25.2)	[98]
South Africa	na	Ag-ELISA	1159	174	15.0 (13.0–17.2)	[98]
Swaziland	na	Ag-ELISA	600	na	28.0 (na)	[99]
Zambia	12/1999-9/2000	Ag-ELISA	628	38	6.1 (4.3–8.2)	[100]

*Abbreviations*: CI, confidence interval; na, not available; IHAT, indirect hemagglutination test

### Taeniosis and bovine cysticercosis occurrence

The co-occurrence of both bovine cysticercosis and taeniosis during the study period was reported in Angola, Ethiopia, Malawi, South Africa, Tanzania, Uganda, Zambia and Zimbabwe, but this was not the case for the other countries/territories studied. The occurrence of bovine cysticercosis or taeniosis was reported for all the countries/territories studied, except for Somalia, Rwanda and the Comoros, Mauritius, Mayotte, Seychelles, Mayotte and Socotra islands.

## Discussion

The present study aimed at describing the epidemiology of *T. saginata* taeniosis/cysticercosis in eastern and southern Africa (1990–2017). Based on our findings, both human taeniosis and bovine cysticercosis were widespread in the 27 countries/territories studied, except for Somalia, Rwanda and six island states/territories, indicating that *T. saginata* is present in most countries of the study area. However, lack of diagnosis and reporting, particularly in rural areas, mean that the data accrued are likely to underestimate occurrence. The absence of data for some countries does not exclude the possibility that this parasite is present there as well. For example, given that of the three countries bordering Rwanda that are included in this review (Burundi, Tanzania and Uganda) all report the presence of this parasite, it seems unlikely that Rwanda is free from *T. saginata*. On the other hand, one potential hypothesis for the lack of reported *T. saginata* in Rwanda is the remarkably higher rate of access to improved sanitation services, at 60.8% in comparison to neighbouring Burundi at 35.5% [38]. The Rwandan civil war, during 1990–1994, culminating in the genocide of 1994, may have impacted reporting during that period, but does not explain the more recent lack of reporting. For Somalia, the ongoing civil war might explain the lack of reported data for the country, whereas for the six island states and territories, governmental or scientific interest in reporting cases may be lacking.

Cases of taeniosis were reported for Angola, Ethiopia, Kenya, Madagascar, Malawi, South Africa, Tanzania, Uganda, Zambia and Zimbabwe, yet the majority of reports on human taeniosis cases did not, unfortunately, provide species determination. Thus, cases of *T. saginata* taeniosis were not differentiated from infections caused by other *Taenia* spp. The pork tapeworm, *T. solium*, for instance, is presumed to also be widely distributed throughout eastern and southern Africa [39] and therefore we cannot conclude that all reported, unspecified taeniosis cases are due to *T. saginata*. *Taenia solium* is known to be the causative agent of the severe condition neurocysticercosis, associated with epilepsy, severe headaches, cognitive deficits [40] and a major cause of deaths among the food-borne diseases [41]. The presence of a single *T. solium* tapeworm carrier poses a major risk for his/her surroundings, as humans acquire neurocysticercosis through the ingestion of *T. solium* eggs transmitted through poor hygiene practices resulting in faecal-oral transmission [42]. Although tapeworm infections usually have an asymptomatic course [7] apart from some sporadic complications (e.g. intestinal obstruction in the Zimbabwean patient [29]), it is thus paramount to register cases as well as to differentiate case species, to allow precise prevalence estimates, and to guide appropriate control measures. Species determination, however, is hampered by the fact that *Taenia* spp. eggs cannot be differentiated upon coprological examination. Expelled proglottids of *T. solium* and *T. saginata* can be distinguished on the basis of the number of uterine branches, but such material is not always available. Moreover, more advanced diagnostic tools (e.g. copro-PCR) to differentiate species are often lacking in resource poor settings [43], and even in developed countries are not often performed due to lack of awareness about neurocysticercosis [12].

In certain countries in the study area, specific culinary habits put the consumers at great risk of contracting *T. saginata* taeniosis. For instance, in Ethiopia, “*kitfo*” is a very popular beef dish, in which the meat is usually consumed raw or lightly cooked, while “*tibs*” is another dish often containing undercooked beef. Furthermore, “*kurt*” refers to the habit of eating cubes of raw beef, finished off with local spices. Unsurprisingly, a high proportion of the Ethiopian population reports having had a tapeworm, and sales of taeniicidal drugs in Ethiopia are high [15, 16, 44–49].

Access to adequate clean water and sanitation services (WASH) is notoriously poor across the whole of sub-Saharan Africa, including the region of interest to this paper. There are large between and within-country disparities, but overall sub-Saharan Africa lags far behind the goals set out by the international community in both the millennium development and the sustainable development goals with only 25.7% (23.1–28.6%) of the population having access to improved sanitation [38]. This lack of WASH capacity is strongly reflected by the presence of parasites such as *T. saginata* which requires ingestion of eggs passed in faecal material for the propagation of its life-cycle.

In eastern and southern Africa the cattle population is large, and bovine products, including meat, are an important protein source for humans, as well as a source of draft power and form of investment. Beef cattle are typically kept in an extensive manner; animals are basically free-ranging. The presence of human *T. saginata* carriers shedding eggs into the environment puts these cattle at risk of bovine cysticercosis, and this presumably occurs widely in the study area. In developed countries, the condition is known to cause economic losses due to freezing or condemnation of the carcass as well as related insurance costs (e.g. Belgium: 3,408,455 EUR/year [13]). Studies investigating the magnitude of this economic loss in the study area are, however, limited, with data available from only one abattoir in Ethiopia [34]. Furthermore, reporting of bovine cysticercosis to OIE appeared to be inconsistent, with large variations in number of cases reported even within the same country, and gaps in the annual reporting (e.g. no data available after 2005).

## Conclusions

*Taenia saginata* taeniosis/cysticercosis is a widespread, yet largely ignored, condition in southern and eastern Africa. This is probably due to the lack of symptoms in cattle, the lack of good data on its economic impact, and because human taeniosis is considered a minor health problem. Nevertheless, the presence of bovine cysticercosis is a clear sign of inadequate sanitation, insufficient meat inspection, and culinary habits that may favour transmission. Measures to reduce transmission of *T. saginata* are therefore warranted, and the infection should be properly monitored, in both humans and cattle. It should also be noted that as cattle are an important source of human protein and livelihoods in the area, ensuring optimal health and productivity of cattle is of indirect importance to human health and welfare as well as any direct impact. Species identification in tapeworm carriers is paramount to gain detailed insights in the distribution of the different *Taenia* spp. in the area, as well to avoid the development of the severe condition neurocysticercosis within communities due to ingestion of eggs shed by a *T. solium* tapeworm carrier. We conclude that in order to ensure both the safety of beef consumed in the southern and eastern Africa, and to improve the underlying sanitary conditions perpetuating the parasitic life-cycle, concerted, co-ordinated efforts must be made by integrating public, animal and environmental health in a One Health approach.

## Additional files

Additional file 1: PRISMA checklist. (DOC 63 kb)

Additional file 2: Search protocol of systematic review. (DOCX 18 kb)

Additional file 3: Databases used in systematic review. (DOCX 16 kb)

Additional file 4: PRISMA flow chart for systematic review. (TIF 1629 kb)

## Abbreviations

ETB: Ethiopian Birr
EUR: Euro
HAART: Highly active antiretroviral therapy
IHAT: Indirect hemagglutination test
OIE: World Organisation for Animal Health/Office International des Epizooties
