# Supplementary material for: Epidemiology of Taenia saginata taeniosis/cysticercosis: a systematic review of the distribution in southern and eastern Africa
Source: Parasit Vectors. 2018 Nov 6;11:578. doi: 10.1186/s13071-018-3163-3 (PMC6219070; doi:10.1186/s13071-018-3163-3)
Supplement: Supplementary file 2 — Search protocol of systematic review. (DOCX 18 kb) [file 13071_2018_3163_MOESM2_ESM.docx]

**Epidemiology of Taenia saginata taeniosis/cysticercosis: a systematic review of the distribution in southern and eastern Africa**

**Search protocol**

**Aim:** To synthesis the current evidence on presence/absence and prevalence (where reported) of *T. saginata* (taeniosis/cysticercosis) in eastern & southern Africa

**Questions to answer:**

Which countries have reported the presence of *T. saginata* in cattle and/or humans since 1990?

For which countries are prevalence data available and what is the quality of that data?

Are specific geographical locations available for these data?

**Methods:**

The review will be conducted in line with the PRISMA statement 2009 (<http://www.bmj.com/content/339/bmj.b2700#alternate>) and will include each item on the PRIMSA checklist <http://www.prisma-statement.org>.

Articles will be selected for inclusion into the systematic review through the identification of all potentially relevant citations through the search strategy. The citations within identified articles will also be included in the screening process. Duplicates will be excluded, followed by screening of titles and abstracts with articles excluded if they do not explicitly report occurrence or prevalence of *T. saginata*. Full text articles will then be screened for exclusion criteria with those remaining utilised in the review.

Full text articles will then be read and relevant data extracted and entered into a Microsoft Excel spreadsheet.

**Databases: See Supplementary Material 2**

**Search term (Pubmed):** (cysticerc* OR cisticerc* OR "C. bovis" OR taenia* OR tenia* OR saginata OR taeniosis OR teniosis OR taeniasis OR ténia OR taeniid OR cysticerque) AND (Angola OR Botswana OR Burundi OR Comoros OR Djibouti OR Eritrea OR Ethiopia OR Kenya OR Lesotho OR Madagascar OR Malawi OR Mauritius OR Mayotte OR Mozambique OR Namibia OR Réunion OR Rwanda OR Seychelles OR Socotra OR Somalia OR South Africa OR Swaziland OR Tanzania OR Uganda OR Zanzibar OR Zambia OR Zimbabwe OR "East Africa" OR "Horn of Africa" OR "Southern Africa" OR Puntland OR Somaliland)

**Inclusion/exclusion:**

- **Exclusion criteria:**
  - studies concerning a different parasite than *T. saginata*
  - studies reporting data from study area
  - studies reporting/using data older than 1990 or published after December 31^st^ 2017
  - studies reporting results out of the scope of the review questions
  - duplicated data
- **Languages:** All
- **Year data collection:** 1^st^ January 1990 – 31^st^ December 2017
- **Geographical range:** All countries/territories within or islands in proximity of eastern & southern Africa
