# Supplementary figures and images for: Epidemiology of Taenia saginata taeniosis/cysticercosis: a systematic review of the distribution in southern and eastern Africa
Source: Parasit Vectors. 2018 Nov 6;11:578. doi: 10.1186/s13071-018-3163-3 (PMC6219070; doi:10.1186/s13071-018-3163-3)

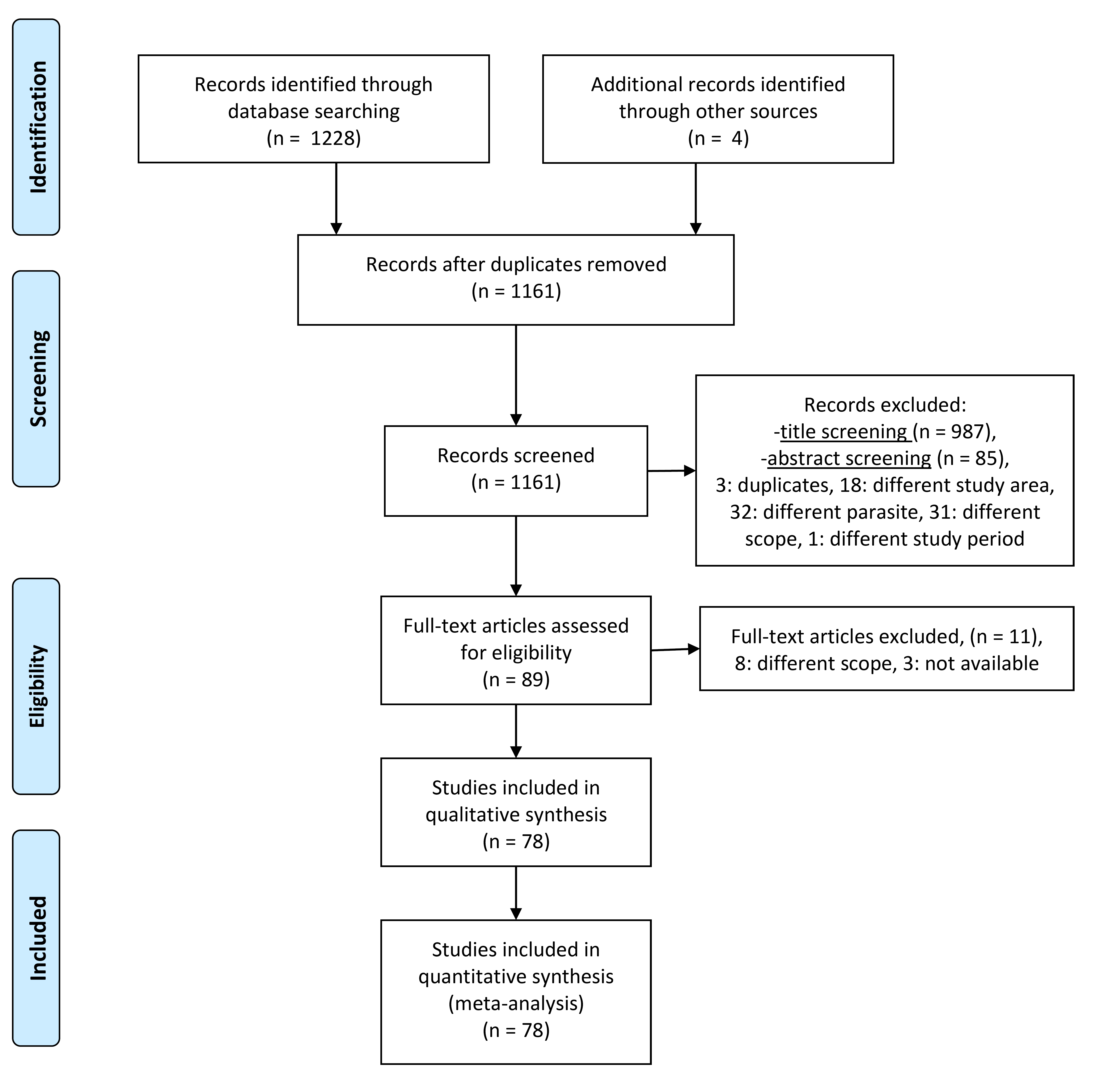

Supplement: Supplementary file 4 — PRISMA flow chart for systematic review. (TIF 1629 kb) [file 13071_2018_3163_MOESM4_ESM.tif]
